# Genetic diversity of *Plasmodium falciparum* in people living with HIV in Ogbomoso, Nigeria: Implications for malaria transmission and treatment

**DOI:** 10.5281/zenodo.15175103

**Published:** 2025-04-08

**Authors:** Adedolapo B. Olorunfemi, Damilola E. Odesola, Ure C. Mbabie, Oreoluwa H. Makinde, Iyanuoluwa O. Olaosebikan, Ogunniran A. James, Adeola O. Ayoola, Oluyinka O. Opaleye, Olusola Ojurongbe

**Affiliations:** 1 Department of Medical Microbiology and Parasitology, Ladoke Akintola University of Technology, Ogbomoso, Nigeria.; 2 Center for Emerging and Re-emerging Infectious Diseases, Ladoke Akintola University of Technology, Ogbomoso, Nigeria.; 3 Department of Evolutionary Anthropology, Duke University, Durham, North Carolina, USA.

## Abstract

**Introduction:**

HIV and malaria coexist in individuals across sub-Saharan Africa, a region profoundly impacted by both diseases. *Plasmodium falciparum* exhibits significant genetic diversity in high-transmission areas, which may further complicate the clinical outcomes of people living with HIV (PLWH). This study investigates the genetic diversity of *P. falciparum* among PLWH in Ogbomoso, Nigeria.

**Materials and Methods:**

A total of 254 blood samples were collected from HIV-positive patients attending clinics at LAUTECH Teaching Hospital and BOWEN Teaching Hospital in Ogbomoso, Oyo State, Nigeria. Malaria infection was diagnosed using a rapid diagnostic test (RDT), microscopy, and nested polymerase chain reaction (nPCR). Genotyping of *msp-1*, *msp-2*, and *glurp* genes was performed to assess genetic diversity. The distribution of allelic families was analysed descriptively using SPSS v.27, and a p-value≤ 0.05 was considered statistically significant.

**Results:**

Among the 254 samples, females (72.8%; mean age 40.7 years) were the most predominant. The prevalence of *P. falciparum* was 5.9%, 55.1%, and 40.6% by RDT, microscopy, and nPCR, respectively. The *msp-1* geno-typing identified 170 distinct variants, with the K1, MAD20, and RO33 families detected at frequencies of 34.7%, 30.0%, and 35.3%, respectively. The *msp-2* genotyping revealed 56 alleles, predominantly from the FC27 family (73.2%). The multiplicity of infection (MOI) for *msp-1*, *msp-2*, and *glurp* genes was 2.02, 1.13, and 1.00, respectively, while the expected heterozygosity (H_E_) values were 0.86, 0.52, and 0.10, respectively. Most *msp-1* (68.5%) and *glurp* (31.1%) samples exhibited polyclonality, whereas *msp-2* samples were predominantly monoclonal (22.1%). .

**Conclusions:**

Despite the high malaria transmission intensity in the region, *P. falciparum* isolates from PLWH exhibited relatively low genetic diversity, suggesting a potential reduction in malaria transmission and signalling the effectiveness of malaria control strategies. Further studies are needed to explore the underlying factors leading to reduced transmission and low genetic variations in this population and their potential impact on malaria transmission and treatment outcomes in PLWH.

## Introduction

Malaria remains one of the most crucial life-threatening parasitic diseases in tropical and subtropical regions [1]. It is caused by *Plasmodium* parasites, which are primarily transmitted through the bites of infected female *Anopheles* mosquitoes [2]. In 2022, Nigeria reported an estimated 68 million malaria cases and 194,000 malaria-related deaths [3]. With one of the highest malaria burdens globally, Nigeria accounts for about 27% of all global malaria cases and 31% of malaria deaths, placing substantial strain on its healthcare system [4]. Among the *Plasmodium* species, *P. falciparum* is the most prevalent and virulent species responsible for malaria in this region [5].

*Plasmodium falciparum* has developed mechanisms such as antigenic switching to evade immune responses, resulting in significant gene diversity. This genetic diversity often complicates the development of effective vaccines and control strategies [6]. Common genetic markers in *P. falciparum* include the merozoite surface protein-1 and - 2 (*msp-1* and *msp-2*) genes, the glutamate-rich protein (*glurp*) gene, and the circumsporozoite protein (*csp*) gene [7]. Among these genetic markers, the *msp-1*, *msp-2*, and the *glurp* genes are widely used to assess allelic diversity, playing a vital role in determining malaria transmission in a community [8]. Their genetic diversity also contributes to *P. falciparum*'s ability to evade the host's immune system and develop resistance to antimalarial drugs. The *msp-1*, *msp-2*, and *glurp* genes are primary targets in the blood-stage malaria vaccine and serve as valuable markers for identifying distinct *P. falciparum* parasite sub-populations [9].

*Plasmodium falciparum* MSP-1, the most abundant merozoite surface protein, is essential for erythrocyte invasion and is associated with malaria severity. This protein, comprising 1,720 amino acids, is organised into 17 blocks with varying levels of conservation [10]. Block 2 of *msp-1* is highly variable, comprising three allelic families: K1, MAD20, and RO33. Similarly, PfMSP-2 is a glycoprotein with a highly polymorphic central repeat region (block 3), flanked by unique variable domains (blocks 2 and 4) and conserved N and C terminal regions. The *msp-2* alleles are classified into two dimorphic families, FC27 and 3D7 [11]. The GLURP protein is composed of a conserved N-terminal non-repeat region covered by amino acids 27–500 (R0 region), a central repeat region with amino acids 500–705 (R1 fragment), and a C-terminal immunodominant repeat region consisting of amino acids 705–1,178 (R2 fragment)[12].

In addition to malaria, Nigeria faces a significant burden of the Human Immunodeficiency Virus (HIV), a viral infection that undermines the immune system by attacking CD4+ T cells, which are essential for immune defence [13]. HIV-induced immunosuppression increases susceptibility to opportunistic infections, including malaria [14]. Among people living with HIV (PLWH), weakened immune responses, coupled with genetic diversity in malaria parasites, may lead to frequent reinfections and prolonged parasitemia. This, in turn, could elevate the risk of severe complications such as cerebral malaria and severe anaemia. Furthermore, parasite genetic diversity might indicate less pressure for drug resistance mutations. The co-infection of malaria and HIV poses a significant public health challenge, especially in regions where malaria is endemic [15]. PLWH have a higher risk of contracting malaria and are more likely to experience severe disease manifestations and complications. Conversely, malaria has been shown to accelerate HIV progression by increasing viral replication rates [16].

Previous studies have explored the association between malaria and HIV in Nigeria [17-18]. However, despite high malaria and HIV prevalence, limited research exists on malaria genetic diversity in PLWH in Nigeria. Understanding the prevalence and genetic diversity of *P. falciparum* in this population is essential and useful for predicting vaccine efficacy and informing malaria control strategies. Thus, this study aims to investigate the genetic diversity of *Plasmodium falciparum msp-1*, *msp-2*, and *glurp* genes in people living with HIV in Ogbomoso, Nigeria.

## Materials and Methods

Sample collection was conducted at the Ladoke Akintola University of Technology (LAUTECH) Teaching Hospital and BOWEN Teaching Hospital in Ogbomoso, Oyo State, Southwest Nigeria. Ogbomoso is located at a latitude of 8° 9' 6.93"N and a longitude of 4° 15' 9.7092"E. The region is characterised by a tropical climate with distinct wet and dry seasons, supporting an environment conducive to malaria transmission due to the abundance of stagnant water sources, which serve as breeding sites for *Anopheles* mosquitoes. Poor waste management and limited access to potable water contribute to the availability of mosquito breeding sites, sustaining malaria transmission in the area. Additionally, inadequate housing structures with poor ventilation and a lack of protective measures, such as insecticide-treated nets (ITNs), exacerbate malaria prevalence.

### Study design and population

This was a cross-sectional, health facility-based study investigating the genetic diversity of *P. falciparum* among HIV patients attending the HIV care center at the study site. Samples were collected between December 2021 and January 2023. The study employed a convenience sampling method for sample collection. Two hundred and fifty-four HIV-positive confirmed individuals who came for regular routine medical checkup at LAUTECH and BOWEN Teaching Hospitals, Ogbomoso were recruited into the study. Patients above 18 years old and who consented to the survey were included in the study. Patients below 18 years, those who had recently used antimalarial drugs within two weeks of sample collection, and those who did not consent were excluded from the study.

### Ethical approval

Ethical approval was obtained from the Ethical Committee, Ladoke Akintola University Teaching Hospital Ogbomoso (Protocol Number: LTH/ OGB/EC/2021/258). The research was carried out following the Code of Ethics of the World Medical Association (Helsinki Declaration) [19]. Informed written or verbal (from uneducated) consent was obtained from all participants.

### Sample collection and malaria diagnosis

Participants were tested for malaria using the Rapid Diagnostic Test Kit (SD BIOLINE Malaria Ag P.f/Pan) during the interview. The test was performed in the field and interpreted within 20 minutes, following the manufacturer's instructions. Dried blood spots (DBS) were collected by finger prick and spotted directly onto labeled 3MM Whatman filter paper. Simultaneously, thick blood smears were prepared for each participant. These smears were examined microscopically to assess malaria parasite density using Giemsa staining. Parasite density was quantified in parasites/μL, based on an assumed average of 8,000 leukocytes per microlitre of blood [20].

The detection limit of light microscopy is 50–100 parasites/μL of blood, and since Polymerase Chain Reaction (PCR) is the most sensitive method (detecting as low as 0.1–1 parasite/μL), discordant cases were classified based on PCR confirmation. A second expert microscopist re-examined a subset of slides to ensure accuracy.

DNA was extracted from DBS on filter paper using the QIAamp Mini DNA extraction kit (QIAGEN, USA), following the manufacturer's instructions. Sixty microliters of DNA were eluted and stored at -20 °C until further analysis.

PCR was conducted on all samples, regardless of microscopy results. Two sets of genus- and species-specific primers (*P. falciparum*, *P. malariae*, *P. ovale*, and *P. vivax*) were used as described previously [21]. DNA was amplified using nested PCR to enable specific species identification across potential sequence variations. All PCR assays were carried out on an Eppendorf™ Mastercycler X50 thermal cycler. The PCR protocol included an initial denaturation step at 95 °C for 5 minutes, followed by 25 cycles (or 30 cycles for the nested PCR) of denaturation at 94 °C for 1 minute, annealing at 60 °C for 2 minutes, and extension at 72 °C for 2 minutes. A final extension was performed at 72 °C for 5 minutes. The PCR products were analysed using gel electrophoresis on a 1.2% agarose gel and visualised under a UV transilluminator. Fragment sizes were compared against a 100 base pair Solis Biodyne DNA ladder.

Amplification of block 2 of *msp-1,* block 3 of *msp-2*, and *glurp* genes was performed using two rounds of nested PCR as previously described [22]. The outer regions of the two genes were initially amplified, followed by a nested PCR with allelic family-specific primer pairs.

For *msp-1*, the polymorphic repetitive region (block 2) was amplified with the following cycling parameters: initial denaturation at 94 °C for 2 minutes, followed by 30 cycles at 94 °C for 30 seconds, annealing at 54 °C (first PCR) or 59 °C (nested PCR) for 1 minute, and extension at 72 °C for 1 minute. A final extension was performed at 72 °C for 5 minutes. Amplification of MSP-2 for both the first and nested PCR followed these conditions: an initial denaturation at 94 °C for 5 minutes (first reaction) and 5 seconds (nested PCR), followed by 35 cycles of 10 seconds at 94 °C, 30 seconds at 57 °C, and 40 seconds at 72 °C. A final extension step was performed at 72 °C for 3 minutes. For the *glurp* gene, a semi-nested PCR reaction targeting the polymorphic R2 region was performed in a final volume of 25 μL using specific primers. The cycling conditions for both reactions were the same, except the annealing temperature for the second reaction was set to 59 °C.

PCR products were analysed via gel electrophoresis on a 1.2% agarose gel and visualised under a UV transilluminator. Fragment sizes were determined relative to a 100 bp DNA ladder using the Azure bioimaging system.

### Data analysis

Data was analysed using SPSS v. 27. For descriptive analysis, proportion was used to present the distribution of different allelic families. The Multiplicity of infection (MOI), defined as the minimum number of distinct genotypes per isolate, was calculated as the mean number of fragments per infected subject in each group. An independent t-test was used to compare the mean MOI according to gender, time of the year of infection, and parasitemia.

The expected heterozygosity (H_E_) was calculated using the following formula:


$$\text{He}=\left[\frac{n}{n-1}\right]\left[1-\sum pi^{2}\right]$$

where n is the number of isolates sampled and pi is the allele frequency at a given locus [23]. A p-value of ≤ 0.05 was considered statistically significant.

## Results

Two hundred fifty-four HIV-positive samples were recruited for the study. Out of the 254 samples, 69 (27.2%) were males, while 185 (72.8%) were females. The overall mean age of participants is 40.7 years. The majority of the participants are secondary school holders (72.1%), traders (44.9%), Christians (70.5%), and married (80.7%) (Table 1).

**Table 1:** Characteristics of participants.

Parameter	Categories	Frequency (%)
Age Group	18-39	87 (34.3)
	40-59	141(55.5)
	60-70+	26 (10.2)
Education	None	2 (0.8)
	Primary	12 (4.7)
	Secondary	183 (72.1)
	Tertiary	57 (22.4)
Occupation	Civil servant	36 (14.2)
	Driver	7 (2.8)
	Student	5 (2.00)
	Trader	114 (44.9)
	Vocational	92 (36.2)
Religion	Christian	179 (70.5)
	Muslim	75 (29.5)
Marital status	Married	205 (80.7)
	Single	19 (7.5)
	Widow	30 (11.8)
Gender	Female	185 (72.8)
	Male	69 (27.2)

### Prevalence of *Plasmodium* species by RDT, Microscopy, and PCR

Using RDT, 15 malaria cases were detected, while microscopy detected 140. The prevalence of *Plasmodium* infection was 5.9% and 55.1% for RDT and microscopy, respectively. Out of the 254 blood samples analysed by nested PCR, 103 (40.6%) samples were confirmed as *P. falciparum*-positive. Twenty-five (9.8%) samples were *P. malariae*-positive, while 20 (7.9%) samples were classified as a mixed infection for *P. malariae* and *P. falciparum*. *Plasmodium ovale* and *P. vivax* were not detected in the samples.

### Demographic characteristics associated with *Plasmodium* infection

The study showed no significant difference between malaria prevalence and all the patients’ demographic characteristics using RDT. In comparison with microscopy and infection with *P. falciparum*, significant differences were observed across all the demographic characteristics. For *P. malariae*, a significant difference was observed for age (p=0.026), education (p=0.042), occupation (p=0.001), and marital status (p=0.001). However, with mixed infection, a significant difference was only observed for occupation (p=0.009) (Table 2).

**Table 2.** Distribution of malaria prevalence in the study population based on social-demographic characteristics of participants.

Baseline Characteristics		RDT +ve (n=15)		Microscopy +ve (n=140)		PCR + ve					
						*P. falciparum (n=103)*		*P. malariae (n=25)*		*P. falciparum+P. malariae (n=20)*	
		n (%)	p-value	n (%)	p-value	n (%)	p-value	n(%)	p-value	n (%)	p-value
Age group	18-39	2 (13.3)	0.120	23 (16.4)	0.004	25 (24.3)	0.001	8 (32.0)	0.026	5 (25.0)	0.117
	40-59	12 (80)		85 (60.7)		62 (60.2)		14 (56.0)		11 (55.0)	
	60-70+	1 (6.7)		32 (22.9)		16 (15.5)		3 (12.0)		4 (20.0)	
Gender	Male	4 (26.7)	0.071	42 (30.0)	0.002	34 (33.0)	0.006	10 (40.0)	0.317	8 (40.0)	0.371
	Female	11 (73.3)		98 (70.0)		69 (67.0)		15 (60.0)		12 (60.0)	
Education	None	1 (6.7)	0.172	-	0.001	4(3.9)	0.001	-	0.042	-	0.135
	Primary	3 (20.0)		16 (11.4)		9 (8.7)		4 (16.0)		-	
	Secondary	7 (46.7)		94 (67.1)		68 (66.0)		14 (56.0)		12 (60.0)	
	Tertiary	4 (26.7)		30 (21.4)		22 (21.4)		7 (28.0)		8 (40.0)	
Occupation	Civil servant	3 (20.0)	0.086	41 (29.3)	0.002	20 (19.4)	0.002	-	0.001	4 (20.0)	0.009
	Driver	-		23 (16.4)		11 (10.7)		2 (8.0)		-	
	Student	2 (13.3)		-		7(6.8)		16 (64.0)		13 (65.0)	
	Trader	8 (53.3)		62 (44.3)		65 (63.1)		5 (20.0)		-	
	Vocational	2 (13.3)		14 (10.0)		-		2 (8.0)		3 (15.0)	
Religion	Christian	9 (60.0)	0.285	93 (66.4)	0.001	64 (62.1)	0.014	17 (68.0)	0.072	17 (85.0)	0.074
	Muslim	5 (33.3)		47 (33.6)		39 (37.9)		8 (32.0)		3 (15.0)	
Marital status	Married	9 (60.0)	0.439	65 (46.4)	0.006	82 (79.6)	0.001	19 (76.0)	0.001	14 (70.0)	0.106
	Single	6 (40.0)		58 (41.4)		13 (12.6)		4(16.0)		6 (30.0)	
	Widow	-		17(12.1)		8 (7.8)		2 (8.0)		-	

### Sensitivity and specificity of RDT and microscopy using PCR as gold-standard

The study showed that RDT had a very high specificity (90.4%) with moderate sensitivity (66.7%), indicating its effectiveness in identifying negative malaria cases correctly but not as effective in identifying positive cases. On the other hand, microscopy was highly sensitive (93.2%) with moderate specificity (75.5%), indicating its effectiveness at detecting true positive cases, but it tended to produce some false positives (Table 3). The moderate sensitivity exhibited by RDT compared to microscopy could be due to the low detection parasite threshold or deletion of *P. falciparum* histidine-rich protein (*pfhrp*)2/3 genes in the RDT. A significant correlation (p<0.0001) was observed between PCR and microscopy sensitivity and specificity. Similarly, there were significant correlations (p< 0.0001) between PCR and RDT specificity, but no correlation was observed with sensitivity.

**Table 3:** Sensitivity and specificity of RDT and microscopy using PCR as the gold standard.

	nPCR		Sensitivity (95% Cl)	P-value	Specificity (95% Cl)	P-value	PPV* (95% Cl)	NPV (95% Cl)
RDT	+ve	-ve						
+ve	10	5	66.7 (42.8-90.5)	0.197	90.4(82.4-98.4)	0.0001	66.8(43.2-44.3)	90.2(74.4-98.2)
-ve	5	234						
Microscopy								
+ve	96	37	93.2(88.3-98.1)	0.0001	75.5(68.6-82.4)	0.0001	72.2(64.6-78.8)	94.2(90.1-98.4)
-ve	7	114						

*PPV: Positive Predictive Value. NPV: Negative Predictive Value. Cl: Confidence Interval

### Distribution of *msp-1*, *msp-2*, and *glurp* genes allelic families among PLWH

Table 4 shows that out of the 103 samples positive for *P. falciparum*, a total of 170 different alleles were identified as *msp-1*. Fifty-nine samples belonged to the K1 (160–280 bp) allelic family, 51 as MAD20 (80–520 bp), and 60 samples to RO33 (160–600 bp) allelic family. The frequency of samples with only K1, MAD20, and RO33 were 34.7%, 30.0%, and 35.3%, respectively. The remaining were polyclonal infections. For polyclonal infections with two allelic types, the frequency of samples with K1/MAD20, K1/RO33, and MAD20/RO33 was 42 (24.7%), 52 (30.6%), and 41 (24.1%), respectively. Thirty-nine (22.9%) isolates were detected to have the three allelic types.

**Table 4:** Distribution and genetic diversity assessment of *P. falciparum* among PLWH.

*P. falciparum genes*	Frequency (%)	Allelic size (bp)	MOI	H_E_	COI	
					Monoclonal (%)	Polyclonal (%)
**MSP-1 (allelic families)**	**170**		**2.02**	**0.86**	**170 (66.9)**	**174 (68.5)**
K1	59 (34.7)	160-280				
MAD20	51 (30.0)	80-520				
R033	60 (35.3)	160-600				
K1/MAD20	42 (24.7)	80-520				
K1/R033	52 (30.6)	160-600				
MAD20/RO33	41 (24.1)	80-600				
K1/MAD20/RO33	39 (22.9)	80-600				
**MSP-2 (allelic families)**	**56**		**1.13**	**0.52**	**56 (22.1)**	**7 (2.8)**
FC27	41 (73.2)	280-550				
3D7	15 (26.8)	80-720				
FC27/3D7	7 (12.5)	80-720				
**Glurp gene**	**50 (38.5)**	**180-980**	**1.00**	**0.10**	**18 (17.5)**	**32 (31.1)**

A total of 56 alleles were identified for *msp-2*, including 41 of FC27 (73.2%) and 15 of 3D7 (26.8%). Allele sizes ranged from 280-550 bp for FC27 and 80-720 bp for 3D7 allelic families. Seven of the isolates (12.5%) were found to be polyclonal, having both *msp-2* allelic families. Fifty isolates were positive for the *glurp* gene (38.5%), with fragment size ranging from 180-980 bp (Table 4).

### Genetic diversity of *msp-1*, *msp-2* and *glurp* gene among PLWH

The multiplicity of infection for *msp-1*, *msp-2*, and *glurp* genes were 2.02, 1.13, and 1.00, respectively. The overall mean multiplicity of infection for *msp-1*, *msp-2* and *gulp* gene is 1.38. The heterozygosity index was 0.86 for *msp-1*, 0.52 for *msp-2*, and 0.10 for the *slurp* gene. The majority of samples that are positive for *msp-1* families (68.5%) and *slurp* genes (31.1%) are polyclonal, while a larger percentage of *msp-2* families exhibited monoclonality (22.1%) (Table 4).

### Association between participant’s age, gender, MOI, H_E_, COI, and *P. falciparum* alleles

Table 5 shows no significant difference between age, gender, MOI, heterozygosity (H_E_), clonality of infection (COI) *msp-1* and *msp-2*. Similarly, no significant difference was observed between age, gender, MOI, and *glurp* gene. However, a significant difference was observed between H_E_ (p=0.002), COI (P=0.048), and the *glurp* gene.

**Table 5:** Relationship between participant’s gender, age, MOI, H_E_, COI, and *P. falciparum* alleles.

Parameters	MSP-1		MSP-2		Glurp	
	n (%)	p-value	n (%)	p-value	n (%)	p-value
Gender		0.43		0.24		0.36
Male	19 (26.8)		15 (27.3)		36 (72.0)	
Female	52 (73.2)		40 (72.7)		14 (28.0)	
Age		0.32		0.72		0.62
18-39	18 (25.4)		12 (21.8)		13 (26.0)	
40-59	43 (60.6)		36 (65.5)		28 (56.0)	
60-70+	10 (14.1)		7 (12.7)		9 (18.0)	
MOI	2.02	0.63	1.13	0.38	1.00	0.18
H_E_	0.67	0.08	0.40	0.41	0.10	0.002
COI		0.21		0.32		
Monoclonal	170 (66.9)		56 (22.1)		18 (17.5)	0.048
Polyclonal	174 (68.5)		7 (2.8)		32 (31.1)	

## Discussion

Co-infection of HIV with malaria poses a substantial global health challenge, especially in malariaendemic regions. Understanding the genetic structure of the malaria parasite population can complement malaria control interventions. This study provides information on the genetic diversity of *P. falciparum* among PLWH in Ogbomoso, Nigeria.

The high prevalence of malaria observed in this study underscores the complexity of malaria epidemiology in the study region. Our previous study [17] conducted among HIV-positive individuals in Osogbo, Nigeria, similarly reported a high prevalence, indicating a consistently high malaria burden among PLWH in this region. The presence of *P. falciparum* or *P. malariae* in the population is noteworthy. Malaria infections caused by these species can lead to intense activation of CD4+ cells and an increased production of proinflammatory cytokines, creating a favourable environment for viral dissemination among CD4+ cells and accelerating HIV-1 replication [24]. Mixed infections (*P. falciparum* + *P. malariae*) may further influence disease severity, treatment response, and immune modulation, particularly in immunocompromised individuals, such as PLWH.

In regions with a high malaria burden, the WHO recommends cotrimoxazole (CTX) prophylaxis for all adults and children living with HIV (both symptomatic and asymptomatic) in Africa. This strategy has demonstrated protective benefits against malaria regardless of CD4 count or clinical stage [25]. However, prolonged exposure of *Plasmodium* species to CTX could reduce its effectiveness, contributing to persistent infections among PLWH. Supporting this concern, a study by Skinner *et al.* [26] found only a modest reduction in malaria parasitemia among patients receiving both CTX and antiretroviral therapy (ART) compared to those on ART alone. Other studies have similarly reported increased malaria incidence in PLWH who discontinued CTX prophylaxis compared to those who maintained it [27–29]. Despite consistent CTX use in our cohort, the sustained high malaria prevalence suggests potential resistance to CTX or suboptimal adherence to prophylactic regimens. Given the shared mechanisms of action and resistance between CTX and sulfadoxine-pyrimethamine (SP), there is growing concern that resistance to one may undermine the efficacy of the other. These findings underscore the need to re-evaluate the long-term effectiveness of CTX prophylaxis in sub-Saharan Africa.

The low RDT positivity in this study could be due to a prozone effect. However, the substantial difference in prevalence rates between RDT and microscopy can be attributed to microscopy's higher sensitivity, particularly for low-density infections. Furthermore, hrp2/3 deletions could contribute to false-negative RDT results as these deletions prevent antigen detection, leading to an underestimation of malaria prevalence. The PCR results demonstrate the value of molecular techniques in accurately identifying infections that may be missed by microscopy or RDT. The analysis revealed no significant differences in malaria prevalence across demographic characteristics when using RDT. However, significant differences were observed with microscopy, particularly in infections caused by *P. falciparum*. This suggests that as the sensitivity of the diagnostic method increases, demographic factors may have a more pronounced influence on malaria prevalence. Additionally, the significant differences observed with microscopy in infections caused by both *P. falciparum* and *P. malariae*, particularly in younger participants, may indicate a shift in transmission dynamics or changes in exposure risk [30]. Similarly, significant differences observed in occupation may reflect varying levels of exposure to malaria vectors, with traders potentially facing higher exposure risks than other occupations. RDT demonstrated high specificity, making it a reliable tool for ruling out malaria in individuals who tested negative. However, its moderate sensitivity and positive predictive value (PPV) indicates that it misses a substantial proportion of true positive cases and produces some false positives. Conversely, microscopy showed higher accuracy in detecting true malaria cases and greater reliability in confirming negative results due to its high sensitivity and negative predictive value (NPV). This suggests that microscopy could serve as an essential diagnostic tool alongside RDT in the study area to ensure true malaria cases are not missed. The significant correlation observed between PCR and microscopy in terms of sensitivity and specificity further supports microscopy as a preferred diagnostic method for detecting malaria parasites in the absence of PCR. This highlights the utility of microscopy for more accurate diagnosis in resource-limited settings.

The genetic diversity of *P. falciparum* plays a crucial role in malaria vaccine development and offers valuable epidemiological insights into transmission intensity within a specific region. The different alleles identified for *msp-1* and *msp-2* in the study showed substantial genetic diversity. The distribution of allelic families (K1, MAD20, and RO33) in *msp-1* indicates a balanced representation of these families in the population. This is contrary to most studies reporting a high prevalence of K1 allelic family [31-32]. The majority of alleles identified for *msp-2* belong to the FC27 family. This is similar to the study conducted in Grande Comore Island [33], where most of the isolates belong to the FC27 family. This dominance suggests spatial dynamics in the genetic profile of *P. falciparum* populations among the study population. Additionally, 38.5% of isolates were positive for the *glurp* gene, indicating a potential reservoir of genetic diversity that may play a role in the parasite's survival and adaptation.

The majority of the patients showed polyclonal infection (having two or more allelic types), which is contrary to the study conducted in south-western Nigeria, where most of the patients showed monoclonal infection [34]. The presence of high polyclonal infections observed with *msp-1* and *glurp* gene is particularly significant, as it may enhance the parasite’s ability to evade the host immune response, complicating the effectiveness of control strategies and the potential for vaccine efficacy. The high number of samples showing multiple alleles underscores the necessity for continuous monitoring of genetic diversity during malaria control efforts.

The MOI for *msp-1* (2.02) suggests a relatively high level of genetic diversity in the parasite population. The genetic diversity observed aligns with high malaria transmission rates in Nigeria and most sub-Saharan African countries [35]. A high MOI of *P. falciparum* has been suggested to increase the risk of clinical outcomes of malaria parasite infections. In contrast, the MOI for *msp-2* (1.13) and *glurp* gene (1.00) indicates a more stable population with fewer distinct alleles per infection. The overall MOI of 1.38 reflects a mixed infection scenario that may influence disease severity and treatment outcomes. Infections with multiple genotypes have been shown to result from high exposure to infective mosquito bites, where parasites are inoculated successively or simultaneously [36]. The H_E_ provides insight into the genetic variability of these alleles, with *msp-1* showing the highest H_E_ (0.86), suggesting significant genetic diversity, which may impact the immune response of individuals. Conversely, *msp-2* (0.52) and *glurp* (0.10) exhibited lower heterozygosity indices, indicating potential challenges for vaccine strategies targeting these alleles. Although extensive genetic diversity is a hindrance to vaccine development efforts, for people living in endemic areas, such diversity enhances the encounter with diverse parasite clones, which in turn, help with the natural acquisition of immune responses against the multiple clones of the parasite and subsequent protection against disease symptoms [37].

The overall low genetic diversity observed in the study population suggests fewer antigenic variants, potentially reducing the parasite’s ability to evade immune detection. In PLWH, HIV-induced immunosuppression already weakens adaptive immunity, meaning malaria infections might persist longer, increasing transmission potential despite lower diversity [38]. In addition, low genetic diversity might indicate less pressure for drug-resistance mutations, suggesting that ACTs may remain effective, reducing the need for urgent drug policy changes. In agreement with this study, research conducted in Mutengene, Cameroon, also reported a low genetic diversity of *P. falciparum* [39].

The lack of significant differences between age, gender, and genetic markers for *msp-1* and *msp-2* indicates that these demographic factors may not significantly influence the parasite's genetic diversity. However, the significant associations observed between H_E_ and COI with the *glurp* gene indicate that genetic diversity in this locus may be influenced by factors not accounted for in the demographic analysis, suggesting a more complex interaction between host and parasite genetics.

This study was restricted to two health facilities in Ogbomoso, Nigeria, limiting the generalisability of findings to broader malaria-endemic regions, particularly those with varying transmission intensities or HIV prevalence rates. Larger-scale studies covering different transmission zones across Nigeria will comprehensively characterise *P. falciparum* genetic diversity and genotype distribution patterns nationwide in PLWH. Additionally, since PCR alone cannot differentiate between alleles with distinct sequences but similar sizes, using advanced molecular techniques, such as next-generation sequencing (NGS), will enable a more precise estimation of distinct alleles and a better assessment of treatment responses in PLWH. Notwithstanding, this study's findings provide valuable insight into malaria burden and genetic diversity in PLWH receiving daily CTX prophylaxis.

## Conclusions

This study demonstrates a low prevalence of *Plasmodium falciparum* and its genetic diversity among PLWH in Ogbomoso, Nigeria. Despite the high malaria transmission intensity in this region, the overall genetic diversity of *P. falciparum* was low. At the *msp-2* loci, most infections are monoclonal, limiting antigenic markers' effectiveness as genotyping tools for tracking drug efficacy.
